# Not so simple: Implementing a sputum jar to enhance TB diagnostic yield

**DOI:** 10.7196/SAMJ.2025.v115i5.3310

**Published:** 2025-06-03

**Authors:** A David, P Marokane, L Singh, J E Farley, P da Silva, W Stevens, L Scott

**Affiliations:** Wits Diagnostic Innovation Hub, School of Pathology, Faculty of Health Sciences, University of the Witwatersrand, Johannesburg, South Africa; National Priority Programme, National Health Laboratory Service, Johannesburg, South Africa; Wits Diagnostic Innovation Hub, School of Pathology, Faculty of Health Sciences, University of the Witwatersrand, Johannesburg, South Africa; School of Nursing, Johns Hopkins University, Baltimore, USA; National Priority Programme, National Health Laboratory Service, Johannesburg, South Africa; Wits Diagnostic Innovation Hub, School of Pathology, Faculty of Health Sciences, University of the Witwatersrand; National Priority Programme, National Health Laboratory Service, Johannesburg, South Africa; Wits Diagnostic Innovation Hub, School of Pathology, Faculty of Health Sciences, University of the Witwatersrand, Johannesburg, South Africa

## To the Editor:

In attempts to reduce sputum specimen rejection rates and improve laboratory workflow and Xpert MTB/RIF Ultra (Cepheid, USA) test accuracy, a modified sputum jar was developed by Sinapi (Sinapi, South Africa (SA)) (https://sinapibiomedical.com/wp-content/uploads12020/09/Specimen-Collection-Cup-IFU-1.pdf). We share lessons learnt on the Sinapi product development lifecycle and not-so-easy implementation roadmap. The Sinapi concept was proposed in 2015 to address sputum specimen rejection rates. This initiated a laboratory descriptive study that reported that of those specimens rejected, the majority were due to leaking specimen containers (61%),^[1]^ which is a biosafety Issue.

The Sinapi prototype design was available in 2017, and by 2019 our laboratory studies demonstrated that the material composition and fabrication did not inhibit standard of care processing (diagnostic molecular testing and liquid culture). Clinical research study implementation commenced in 2021 (during the COVID-19 pandemic) at a tertiary hospital in Gauteng Province, SA, with several hurdles encountered, as outlined in Fig. 1.

It became apparent that the sputum specimen journey post collection to laboratory referral (known as the pre-analytical phase) was not standardised across the region. Despite this, and although 59 cups could not be accounted for, the introduction of Sinapi cups resulted in a lower sputum rejection rate of 1.7% (13/772), with no rejection attributed to specimen leakage. In addition, we performed a feasibility study among healthcare and laboratory workers that generated a medium system usability score of 83 (good performance).

Overall, we learnt that although early field evaluations inform product improvements, a multi-prong approach with on-the-ground ongoing training, key opinion leader engagement and complete specimen journey data collection are integral to translational research. As Broger *et al*.^[2]^ highlight, the importance of diagnostic yield (the proportion of people in whom a diagnostic test identifies tuberculosis (TB) among all people for whom testing is attempted) will be key for investigating future TB diagnostic strategies. In addition, reducing rejections will reduce loss to follow-up. We hope that our lessons learnt on simple sputum jars will be valuable to the new specimen collection strategies such as tongue swabs, and ensure that processes are developed and implemented to support the entire diagnostic value chain.

## Figures and Tables

**Fig. 1.**
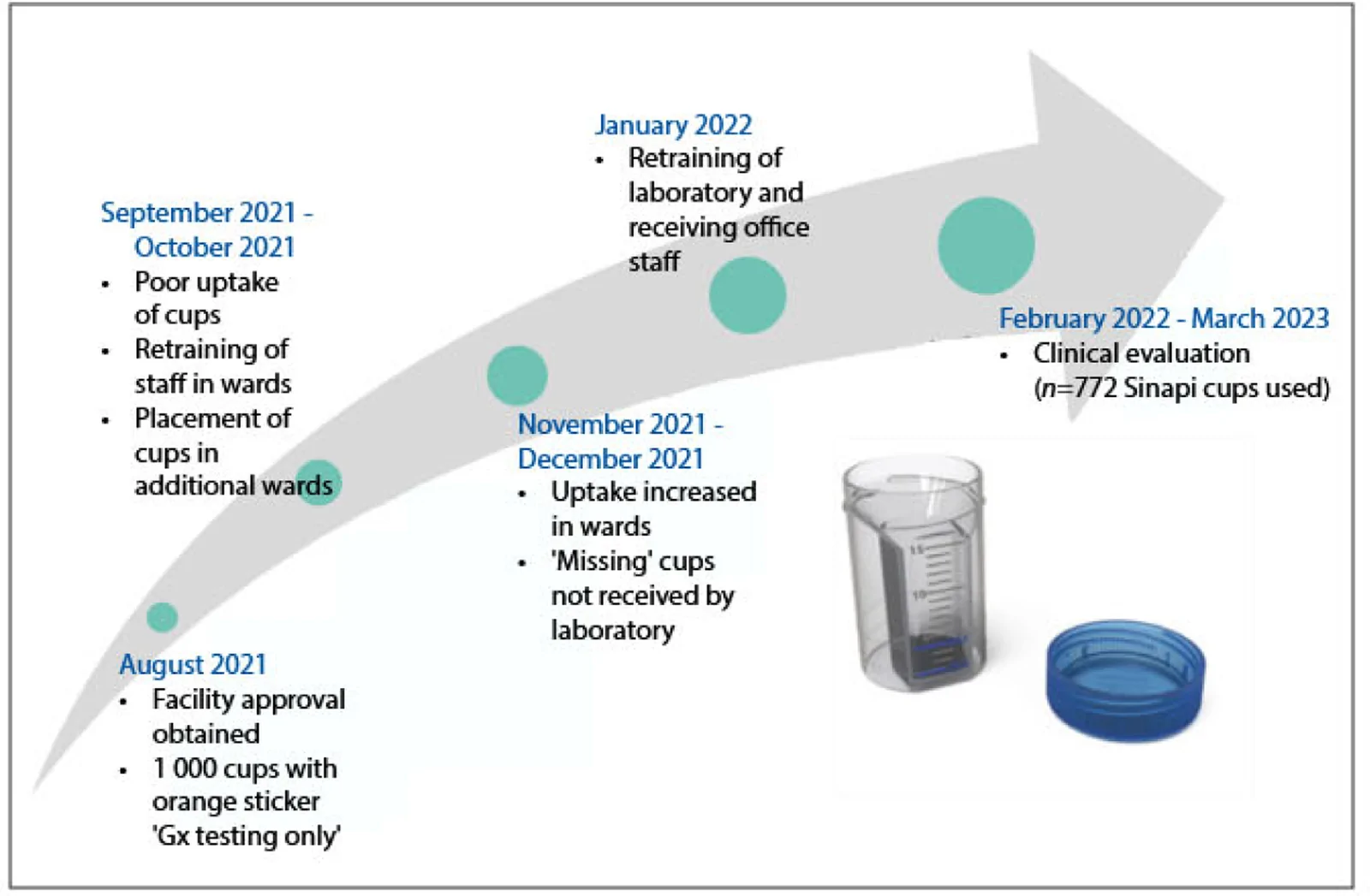
Sinapi cup clinical implementation timelines and challenges experienced. (Gx = GeneXpert (xpert MTB/RIF Ultra).)

## Data Availability

The data supporting this study’s findings are available from the corresponding author, AD, upon reasonable request and upon National Health Laboratory Service institutional approval.

## References

[1] Zerbini MS, Singh S, Botha M, Ghebrekristo Y, Opperman CJ. Specimen rejection in a high-throughput TB laboratory: A descriptive study. S Afr Med J 2023;113(10):e1364. 10.7196/SAMJ.2023.v113i10.1364 37881905

[2] Broger T, Marx FM, Theron G. Diagnostic yield as an important metric for the evaluation of novel tuberculosis tests: Rationale and guidance for future research. Lancet Glob Health 2024;12(7):e1184–e1191. 10.1016/S2214-109X(24)00148-7 38876764

